# 10-year clinical outcomes of subthalamic nucleus versus pallidal deep brain stimulation for Parkinson’s disease: VA/NINDS CSP #468F

**DOI:** 10.3389/fneur.2025.1728999

**Published:** 2026-01-16

**Authors:** Jill L. Ostrem, Ping Luo, Frances M. Weaver, Kenneth Follett, Johannes Rothlind, Nicholas B. Galifianakis, Eugene C. Lai, Jeff Bronstein, John Duda, Kathryn Holloway, Aliya Sarwar, Matthew Brodsky, Kathryn Chung, Meredith Spindler, Domenic Reda, Amanda Snodgrass, Claudia Moy, Grant Huang, Yongliang Wei, William J. Marks, the CSP 468F Study Group

**Affiliations:** 1 Department of Neurology, University of California, San Francisco, San Francisco, CA, United States; 2 San Francisco VA Medical Center, San Francisco, CA, United States; 3 Edward Hines Jr, VA Hospital, Hines, IL, United States; 4 Department of Neurosurgery, University of Nebraska Medical Center, Omaha, NE, United States; 5 Department of Neurology, Houston Methodist Neurological Institute, Houston, TX, United States; 6 Department of Neurology, University of California, Los Angeles, Los Angeles, CA, United States; 7 West Los Angeles VA Medical Center, Los Angeles, CA, United States; 8 Department of Neurology, Philadelphia VA Medical Center, Philadelphia, PA, United States; 9 McGuire VA Medical Center, Richmond, VA, United States; 10 Department of Neurosurgery, Virginia Commonwealth University, Richmond, VA, United States; 11 Michael E. DeBakey VA Medical Center, Houston, TX, United States; 12 Department of Neurology, Baylor College of Medicine, Houston, TX, United States; 13 Department of Neurology, Oregon Health Science University, Portland, OR, United States; 14 Portland VA Medical Center, Portland, OR, United States; 15 Department of Neurology, University of Pennsylvania, Philadelphia, PA, United States; 16 VA Cooperative Studies Program Clinical Research Pharmacy Coordinating Center, Albuquerque, NM, United States; 17 The National Institutes of Neurological Disorders and Stroke, Bethesda, MD, United States; 18 The Department of Veterans Affairs Cooperative Studies Program Central Office, Washington, DC, United States; 19 Department of Neurology, Stanford University School of Medicine, Stanford, CA, United States

**Keywords:** deep brain stimulation, globus pallidus, long-term outcomes, Parkinson’s disease, subthalamic nucleus

## Abstract

**Objective:**

This study aimed to examine the very long-term effects of globus pallidus interna (GPi) or subthalamic nucleus (STN) deep brain stimulation (DBS) on Parkinson’s disease (PD) in a subset of patients enrolled in the CSP468 VA/NINDS prospective randomized trial.

**Methods:**

The primary outcome was the change in off-medication/on-stimulation Unified Parkinson’s Disease Rating Scale III score (UPDRS III) from baseline over time between the two targets at 2, 7, and 10 years. Many secondary outcomes were also explored.

**Results:**

A total of 156 patients were enrolled in this substudy, and data were available for 68 GPi/49 STN participants at 7 years and 49 GPi/28 STN participants at 10 years. There was no overall difference in the time trend between the two targets (*p* < 0.09). UPDRS III improvements from baseline in the GPi cohort at 2, 7, and 10 years were 39.9% (*p* < 0.001), 16.4%, (*p* < 0.001), and 22.3%, (*p* = 0.10), respectively, and in the STN cohort at 2, 7, and 10 years it was 34.9% (*p* < 0.001), 16.9%, (*p* < 0.001), and 32.8%, (*p* < 0.001), respectively. Tremor subscores showed the greatest reduction, followed by rigidity subscores. Initial improvements in bradykinesia and axial subscores were attenuated, and UPDRS I, II, and III on-medication/on-stimulation scores significantly declined. UPDRS IV scores and motor diaries showed significant long-term improvement, and medication reductions were seen regardless of the target. At 7 and 10 years, the PDQ-39 total score no longer showed improvement, and more severe cognitive impairment was seen in both targets.

**Conclusion:**

DBS therapy has a significant beneficial effect on overall motor function, dyskinesia, and motor fluctuations over 10 years (regardless of target), though non-motor symptoms progressed. Bradykinesia, axial, and quality-of-life improvement were maintained at 2 years and then declined over time.

**Clinical trial registration:**

ClinicalTrials.gov, identifier NCT01022073, NCT00056563, NCT01076452.

## Introduction

Deep brain stimulation (DBS) is a highly effective therapy for Parkinson’s disease (PD) patients experiencing motor complications inadequately addressed by pharmacotherapy. Numerous randomized controlled trials have been published describing remarkable outcomes after 6–12 months of DBS therapy compared to best medical therapy (1–6). A number of well-conducted open-label trials within a given brain target have demonstrated robust motor outcomes after 3–5 years of DBS (7–13). Many studies also now document even longer-term outcomes (8–17 years), describing the experience with the subthalamic nucleus (STN) target (14–22) or, in far fewer studies, with the globus pallidus internus (GPi) target (23, 24). Most studies consistently reported overall motor benefit with a decline in axial symptoms and postural instability, improvement in levodopa-related complications, medication requirements less than pre-DBS baseline, more cognitive impairment, and reduced global benefit due to disease progression (14–16, 18–25).

The longstanding debate of the “best” stimulation target for PD treatment between the subthalamic nucleus (STN) and globus pallidus internus (GPi) started with early uncontrolled European studies, which suggested the durability of GPi DBS might be inferior to STN DBS (26–28). This included a 5-year follow-up study published in 2004 describing a progressive decline in benefit in 11 GPi DBS PD patients, 4 of whom went on to subsequently be treated with bilateral STN DBS and obtained further improvement (29). Since then, much has been learned about the two targets, with countless small, open-label, non-randomized studies of STN versus GPi DBS (30, 31) and several prospective, randomized, controlled studies and meta-analyses reporting generally mixed or similar overall motor benefit at short and intermediate time points after treatment (25, 31–37). Two of these randomized trials have published 3-year outcomes. Odekerken et al. reported a slight motor advantage in off-phase motor improvement with STN DBS, but a similar risk of cognitive, mood, and behavioral complications at 36 months for both targets (38). The original CSP 468 VA/NINDS trial showed no significant motor differences between the targets and no group differences in cognitive outcomes, except for a greater 3-year decline on the Mattis Dementia Rating Scale (MDRS) for STN than GPi patients (39). A published meta-analysis investigated the results of STN versus GPi DBS in randomized trials at varying lengths of follow-up time, concluding that the motor benefits achieved with GPi and STN DBS in PD were similar; STN DBS allowed for greater medication reduction, and GPi DBS favored mood outcomes (36).

There are no published studies from prospective, randomized trials that examine outcomes beyond 3 years comparing STN DBS to GPi DBS. Here, we present the extended long-term outcomes after DBS—out to 10 years in some patients—from the CSP 468 VA/NINDS cohort.

## Materials and methods

### Participants

This multi-center, longitudinal follow-up study included PD participants enrolled in the CSP#468 VA/NINDS Cooperative trial treated with bilateral GPi or STN DBS. Among 299 participants randomized to the DBS target in CSP#468, 290 received DBS surgery between June 2002 and November 2006, 279 participants completed the 6-month visit evaluations, and 261 participants completed the 2-year visit evaluations (33).

**Figure 1:**
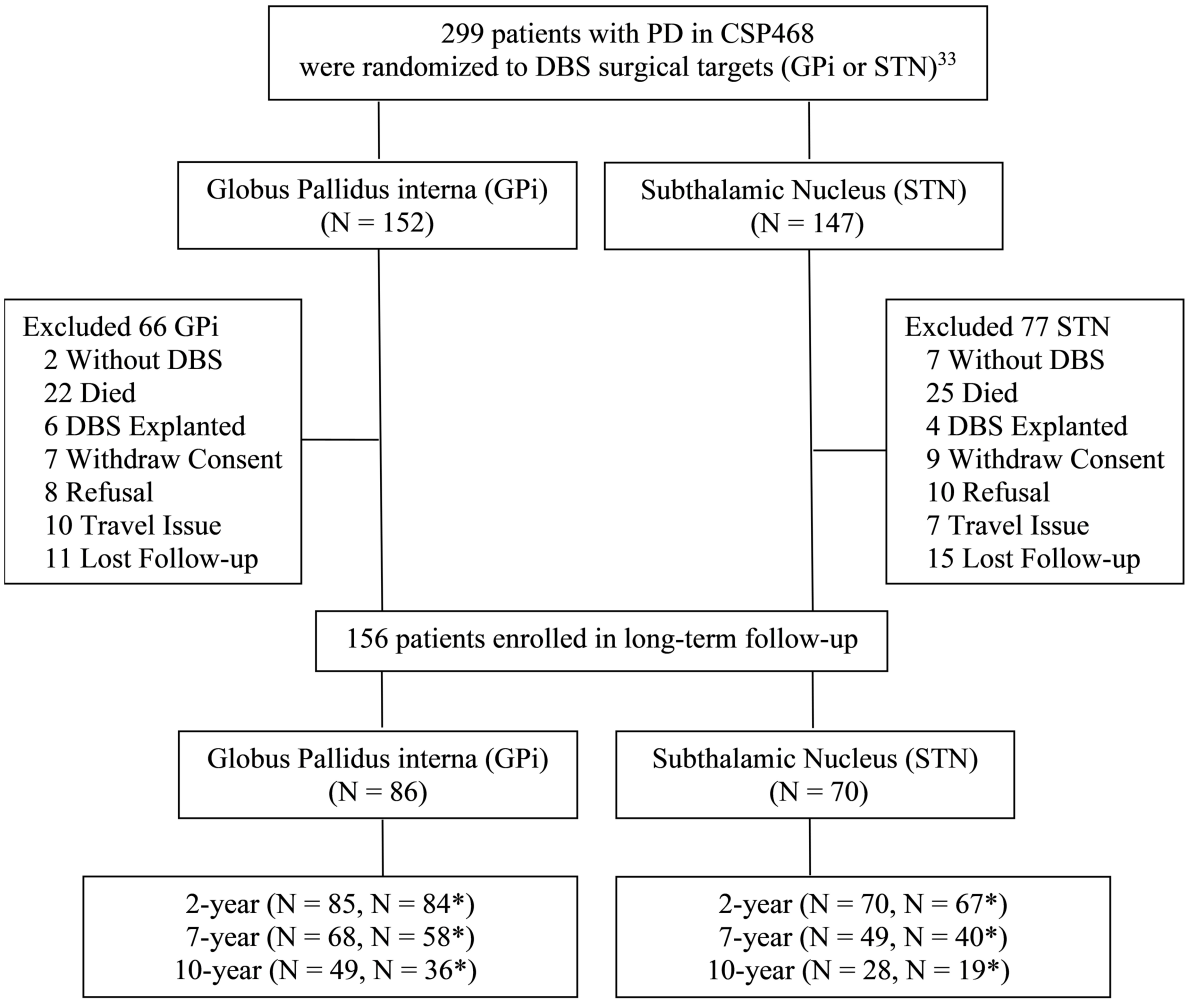
Study flow-chart. *Patients with UPDRS III off mediation/on stimulation (primary outcome).

The main study ended in 2008, and this long-term extension study started in 2010. All eligible participants who were enrolled in VA CSP#468 at VA Parkinson’s Disease Research Education and Clinical Centers (PADRECCs) and affiliated university sites who were still alive were re-screened for participation in this extension CSP#468F long-term follow-up study (Figure 1). Participants were excluded from enrollment into the follow-up study if they (1) had died, (2) had their DBS permanently explanted or turned off, (3) were lost to follow-up (unable to contact), (4) refused to participate, or (5) had previously withdrawn consent. As part of this process, a review of patient electronic medical records and Medicare databases was performed to capture serious medical events occurring since the CSP#468 primary study ended to help determine participants’ eligibility.

All site institutional review boards approved the study, and participants provided written informed consent [clinicaltrials.gov identifiers: NCT00056563 (2), NCT01076452 (33), and NCT01022073].

### Clinical assessments

Participants were evaluated locally in person every 12 months (± 30 days) using a standardized protocol, resulting in follow-up out to 10 years from the original implant in some patients. The primary outcome measure was the change in the off-medication/on-stimulation Unified Parkinson’s Disease Rating Scale (UPDRS) (40) motor score (part III) at 2, 7, and 10 years of treatment, compared to baseline, between the two targets. Multiple secondary outcome measures included off-medication/on-stimulation UPDRS III subscores (tremor, rigidity, bradykinesia, and axial), on-medication/on-stimulation UPDRS-III motor score, and UPDRS I, II, and IV with Hoehn and Yahr staging and Schwab and England (S&E) scoring (41); 24-h 1-day motor diary (completed within the week prior to visit) that characterized time into “on time without troublesome dyskinesia,” “on time with troublesome dyskinesia,” “off time,” and “asleep”, the PDQ-39 (42), and the Beck Depression Inventory (43). A subset of the measures included in the baseline neuropsychological assessment battery was administered annually to participants who earned raw scores of 100/144 or higher on the Mattis Dementia Rating Scale (MDRS) (44) (data not presented here). Study nurses and coordinators were pre-trained by study neuropsychologists to assist in the completion of these measures for this longitudinal follow-up. Other data collected included documentation of DBS stimulation parameters and device diagnostics; device issues; medication use defined as daily levodopa equivalent dose; and serious adverse events (SAEs) related to the DBS device, DBS surgery, or PD progression. If the participant could not be evaluated in person every 12 months due to health status and/or travel burden, then a phone visit collected demographic and medical events, UPDRS I, UPDRS II, UPDRS IV, motor diary, PDQ-39, PD medications, and stimulation parameters.

### Statistical analysis

All analyses were based on the intent-to-treat principle by maintaining the randomized group assignments from the original study. *T-tests* or Fisher’s exact tests were used for comparisons of baseline characteristics between treatment groups. Medication was converted to levodopa equivalents for analysis (33). Mixed-effects analyses were conducted for repeated measurements over time, treating the missing data as missing at random. Time intervals were treated as categorical variables in analyzing the changes over time or between any pair of two time points. Hypothesis tests with orthogonal polynomial contrasts were applied to the time trend effect and its interaction with the treatment group. Analyses were performed using SAS software (version 9.4). All statistical tests were two-sided, and a *p-*value of 0.05 was considered statistically significant with no formal correction for multiple analyses.

## Results

### Patient characteristics

A total of 156 patients (86 GPi/ 70 STN) were enrolled from the original study into this long-term follow-up study; this included 68 GPi and 49 STN participants who completed visits at 7 years, and 49 GPi and 28 STN participants who completed study visits at 10 years. Some patients did not complete the full UPDRS III off-medication testing at 7 and 10 years, which reduced the number of patients contributing to the primary outcome (7 years: 58 GPi and 40 STN; 10 years: 36 GPi and 19 STN participants) (Figure 1). An analysis comparing secondary outcome measures for those who completed and did not complete the primary outcome suggested that overall the subgroup of patients not completing the UPDRS III off-medication testing was likely more advanced. At year 10, the UPDRS II activities of daily living score and PDQ mobility, ADL, emotional wellbeing, and communication subscores (but not other secondary measures) were all statistically worse (*p* < 0.05) in this subgroup.

Baseline (pre-DBS) characteristics for patients enrolled in this follow-up study are summarized in Table 1, and no significant differences were noted between STN and GPi cohorts. Given the high dropout rate over time, we also compared baseline characteristics for the subgroups of patients completing year 7 and 10 visits and did not find any differences between the 2 target groups. However, by comparing the 156 patients who entered this sub-study to the 299 who enrolled in the original study, we found the long-term participants were generally “healthier” than the original trial population. Overall, at baseline, those with extended follow-up were younger (*p* ≤ 0.001) had lower H&Y off-medication scores (*p* ≤ 0.05), and had higher MDRS scores (*p* ≤ 0.01). Patients enrolled in the long-term follow-up also had higher *S&E* off-med scores (*p* ≤ 0.001), lower UPDRS I (*p* ≤ 0.001), II (*p* ≤ 0.001), and III scores (*p* ≤ 0.05), lower PDQ-39 single index scores (*p* ≤ 0.001), and higher processing speed index scores (*p* ≤ 0.01) as measured at 2-year follow-up.

**Table 1:** Baseline pre-DBS characteristics for CSP 468F enrolled patients.

Results are presented in *N* (%) or mean (SD)	GPi (*N* = 86)	STN (*N* = 70)	*P*-value
Age (years)	59.5 (8.4)	58.6 (7.9)	0.51
Age (≥ 70 years)	11 (12.8%)	7 (10.0%)	0.62
Gender—male	75 (87.2%)	58 (82.9%)	0.50
Race—white	84 (97.7%)	66 (94.3%)	0.26
Married	65 (75.6%)	49 (70.0%)	0.50
Living with family	75 (87.2%)	57 (81.4%)	0.39
Education (years)	14.3 (3.2)	15.3 (3.2)	0.07
Years since PD diagnosis	11.4 (4.7)	11.4 (5.2)	0.96
Years on PD Medications	11.0 (4.7)	10.6 (4.7)	0.57
Levodopa equivalent PD medication (mg)	1,330 (585.9)	1,283 (516.9)	0.59
UPDRS I (mentation, behavior, and mood)^a^	2.5 (2.0)	2.6 (2.1)	0.70
UPDRS II (activities of daily living)^a^	18.2 (5.3)	18.9 (6.4)	0.44
UPDRS III (motor function off-medication)^a^	43.2 (12.1)	43.2 (13.9)	0.99
UPDRS III (motor function on-medication)^a^	22.3 (11.2)	21.1 (9.6)	0.51
UPDRS IV (complication of therapy)^a^	8.6 (2.8)	9.2 (3.2)	0.21
Hoehn and Yahr scale (off medication)	3.2 (0.9)	3.3 (0.8)	0.43
Schwab and England scale (off medication)	52.0 (21.4)	52.9 (18.4)	0.79
Motor function without dyskinesia hours per day	6.6 (2.7)	7.0 (2.9)	0.36
Motor function with troublesome dyskinesia	4.3 (3.5)	4.3 (3.3)	0.92
PDQ-39 single index (range 0–100)^a^	42.4 (14.4)	47.3 (12.5)	0.03
PDQ-39 mobility (range 0–100)^a^	54.1 (23.3)	59.4 (19.5)	0.13
PDQ-39 ADL (range 0–100)^a^	54.2 (17.5)	55.3 (18.3)	0.70
PDQ-39 emotional wellbeing (range 0–100)^a^	37.9 (19.8)	42.9 (17.7)	0.10
PDQ-39 stigma (range 0–100)^a^	41.0 (24.7)	44.2 (26.0)	0.44
PDQ-39 social support (range 0–100)^a^	23.8 (17.3)	30.7 (19.0)	0.02
PDQ-39 cognition (range 0–100)^a^	38.5 (16.6)	43.1 (15.8)	0.08
PDQ-39 communication (range 0–100)^a^	41.2 (19.8)	49.4 (19.5)	0.01
PDQ-39 bodily discomfort (range 0–100)^a^	47.8 (21.8)	51.8 (21.0)	0.26
Beck Depression Inventory (range 0–63)^a^	10.7 (8.6)	10.4 (6.6)	0.87
Mattis dementia scale total (range 0–144)^b^	138.2 (5.0)	138.2 (4.8)	0.96

PD, Parkinson’s disease; UPDRS, Unified Parkinson’s Disease Rating; PDQ-39, Parkinson’s Disease Questionnaire-39 Score.

a Higher score indicates worse functioning.

b Higher score indicates better functioning.

### Clinical outcomes

**Figure 2:**
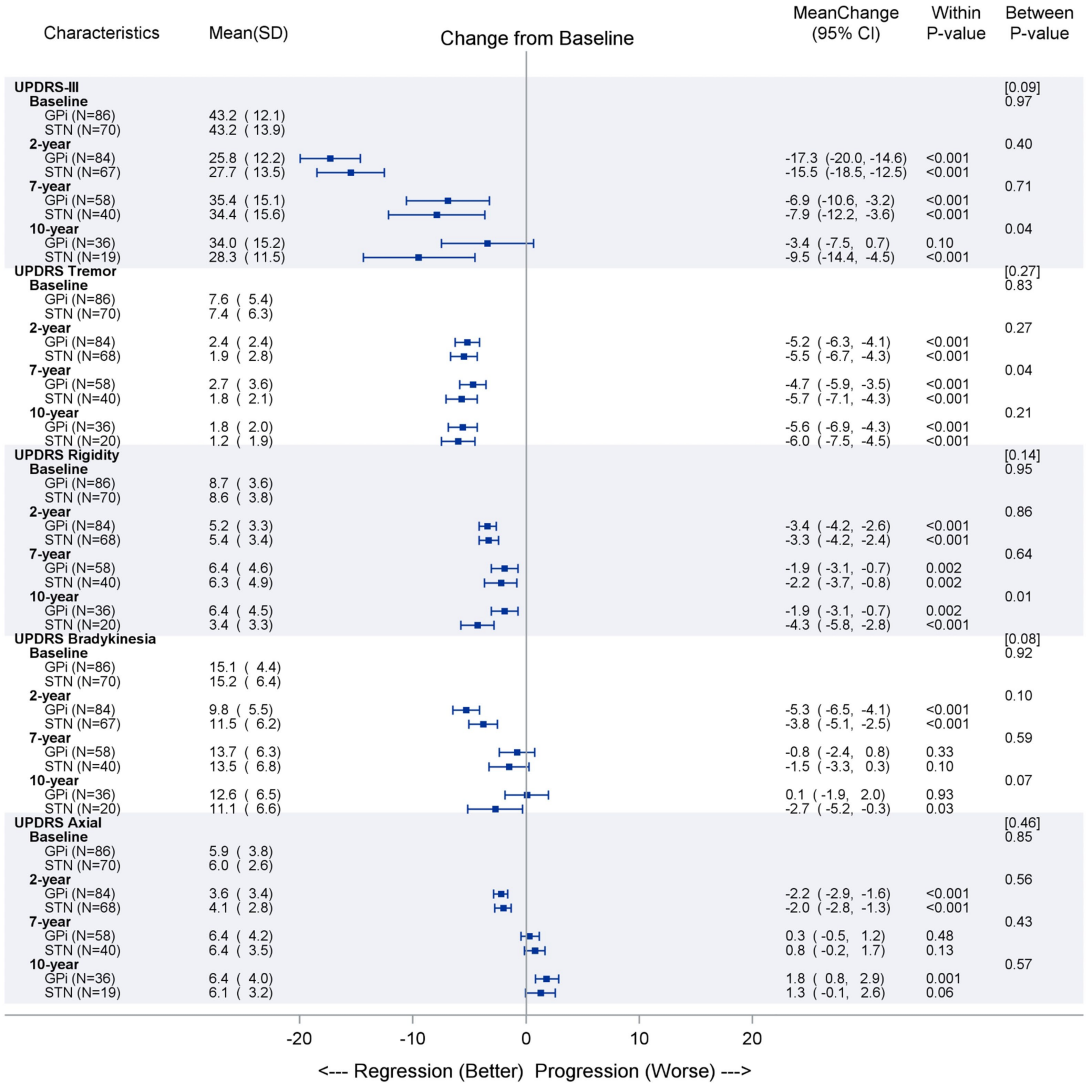
Primary outcome (UPDRS part III off medication/on stimulation) over time. The observed data are described as mean (SD) separately by GPi and STN groups at each time point for primary outcome UPDRS III (total score and 4 subscales). The mean of changes from baseline to later time points are reported together with the 95% confidence interval and illustrated as dot and error bars in the plot. The *p*-values were obtained for either the changes from baseline within each group or the between-group comparisons at each time point. The overall linear time trends between two groups are compared and the *p*-value are reported in brackets above those between-group *p*-values. Subscores calculated from summation of items as follows: Tremor score items 20 and 21; Rigidity score corresponding to the sum of item 22; Bradykinesia score corresponding to the sum of item 23, 24, 25, and 26; Axial score used UPDRS-III scores corresponding to the sum items 27, 28, 29, and 30.

The UPDRS III off-medication/on-stimulation scores reflected improvement in motor function compared to baseline at 2, 7, and 10 years for GPi (43.2 to 25.8, *p* < 0.001; 35.4, *p* < 0.001; and 34.0, *p* = 0.10) and STN (43.2 to 27.7, *p* < 0.001; 34.4, *p* < 0.001; and 28.3, *p* < 0.001), respectively (Figure 2). There was no overall difference in time trend over time between the two targets (*p* < 0.09). The tremor subscores showed the greatest reduction over time (*p* < 0.001), followed by rigidity subscores (*p* ≤ 0.002), regardless of target. There was a significant difference between GPi and STN for rigidity (favoring STN), but only at 10 years (*p* < 0.01). Bradykinesia and axial subscores showed improvement at 2 years (*p* < 0.01), but then effectiveness is attenuated over time for both targets.

**Figure 3:**
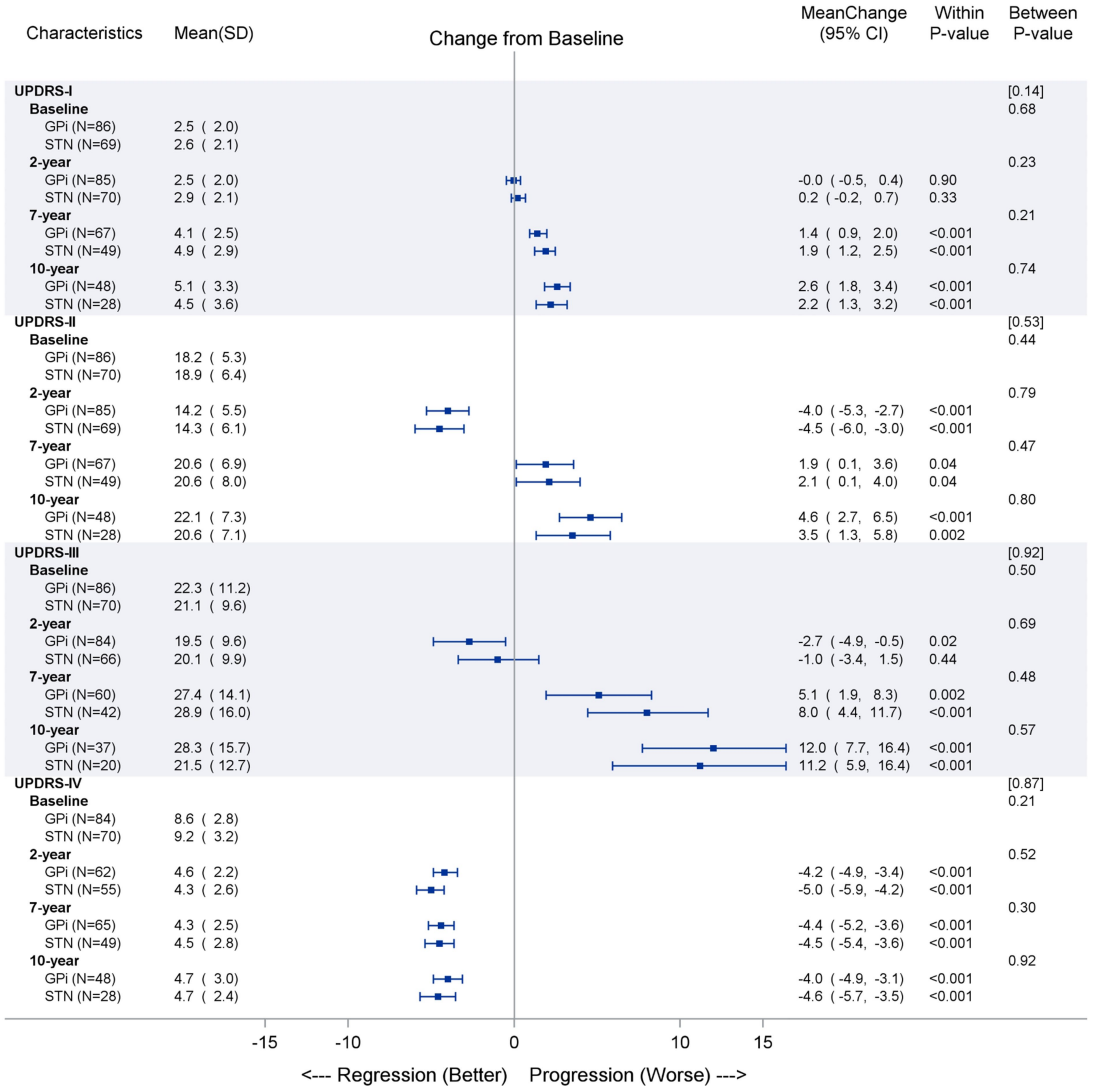
UPDRS secondary outcomes (UPDRS I, II, III on medication, and IV) over time. The observed data are described as mean (SD) separately by GPi and STN groups at each time point for UPDRS on-medication and on-stimulation outcomes (part I, II, III, and IV). The mean of changes from baseline to later time points are reported together with the 95% confidence interval and illustrated as dot and error bars in the plot. The *p*-values were obtained for either the changes from baseline within each group or the between-group comparisons at each time point. The overall linear time trends between two groups were compared and *p*-value are reported in brackets above those between-group *p*-values.

Changes in UPDRS I, II, III on meds, and IV scores are shown in Figure 3 and reflect a similar pattern at 2-year post-surgery as previously reported in the original study (33). Both targets showed no improvement in UPDRS I from baseline at 2 years and then a significant worsening in UPDRS I at 7 and 10 years (*p* < 0.001). At 2 years, both targets showed significant improvements in UPDRS II and UPDRS III on medication scores (*p* < 0.001), but at years 7 and 10, a worsening from baseline was seen (*p* < 0.04 and *p* < 0.001, respectively). On average, a calculated “gait score” (summation of UPDRS II on medication item 13 (falling unrelated to freezing), item 14 (freezing when walking), and item 15 (walking) (45) showed improvement in both targets at 2 years (GPi 4.0 to 3.3, *p* ≤ 0.003 and STN 4.6 to 3.4, *p* < 0.001) but worsened at year 7 (GPi 5.2, *p* < 0.001 and STN 4.9, *p* = 0.18) and year 10 (GPi 5.6, *p* < 0.001 and STN 4.9, *p* = 0.26) with fewer changes seen with the STN target. The UPDRS IV improvement was not different between targets and continued to be significantly improved at 2, 7, and 10 years (*p* < 0.001).

Table 2 highlights the motor diary, PDQ-39, and Beck Depression Inventory data. Remarkable sustained improvements were seen in on-time without troublesome dyskinesia, reduced time with troublesome dyskinesia, and reduced off-time regardless of target at 2, 7, and 10 years (*p* < 0.001). The PDQ-39 single index score found significant improvement in PD quality of life at 2 years, regardless of target (*p* < 0.001), but a return to near baseline scores at 7 and 10 years. There were no significant differences between targets on PDQ-39 subscores, and improvements observed at 2-year post-surgery for these subscores were attenuated during the long-term follow-up. Repeated administration of the Beck Depression Inventory yielded comparable results between targets regarding self-ratings of mood.

**Table 2:** Motor diary, PDQ-39, Beck Depression Inventory, and medication outcomes.

Secondary outcomes	GPi-DBS^a^				STN-DBS^a^			
	Baseline (*N* = 86)	2-year (*N* = 85)	7-year (*N* = 68)	10-year (*N* = 49)	Baseline (*N* = 70)	2-year (*N* = 70)	7-year (*N* = 49)	10-year (*N* = 28)
Motor function diaries								
Good motor function/on time without troublesome dyskinesia (hours/day)	6.6 ± 2.7	12.3 ± 4.0**	10.7 ± 3.9**	10.5 ± 3.6**	7.0 ± 2.9**	12.3 ± 4.0**	11.4 ± 4.0**	11.1 ± 4.4**
On time with troublesome dyskinesia (hours/day)	4.3 ± 3.5	1.3 ± 2.7**	1.2 ± 2.5**	1.1 ± 2.1**	4.3 ± 3.3**	1.0 ± 2.0**	0.7 ± 1.3**	1.6 ± 2.8**
Poor motor function/off time (hours/day)	5.8 ± 2.5	2.4 ± 2.8**	3.2 ± 3.8**	3.3 ± 3.0 **	5.9 ± 3.1**	2.7 ± 4.0**	2.5 ± 2.6**	2.6 ± 3.0**
Asleep (hours/day)	7.2 ± 1.9	8.0 ± 1.9	8.3 ± 2.1	8.9 ± 1.9	6.8 ± 1.9	7.9 ± 1.9	8.9 ± 2.3	7.8 ± 2.6
Parkinson’s disease questionnaire-39 (range 0–100)^c^								
Single index	42.4 ± 14.4	34.7 ± 14.9**	42.0 ± 14.0	47.3 ± 15.2*	47.3 ± 12.5	37.7 ± 14.4**	44.9 ± 15.1	47.0 ± 17.4
Mobility	54.1 ± 23.3	39.1 ± 23.9**	59.7 ± 26.8	65.6 ± 26.1**	59.4 ± 19.5	44.1 ± 22.0**	61.2 ± 27.3	66.5 ± 30.3
Activities of daily living	54.2 ± 17.5	37.2 ± 18.7**	52.1 ± 23.1	56.2 ± 25.2	55.3 ± 18.3	37.7 ± 20.6**	50.3 ± 27.7	53.3 ± 26.7
Emotional role function	37.9 ± 19.8	32.7 ± 20.4*	38.8 ± 20.3	43.4 ± 18.2	42.9 ± 17.7	35.0 ± 19.4**	44.1 ± 22.6	39.9 ± 26.2
Stigma	41.0 ± 24.7	26.9 ± 21.1**	28.8 ± 23.6**	26.2 ± 22.1**	44.2 ± 26.0	28.2 ± 24.9**	30.3 ± 28.0**	30.6 ± 27.3**
Social support	23.8 ± 17.3	24.1 ± 18.4	21.5 ± 17.7	25.9 ± 18.2	30.7 ± 19.0	29.3 ± 19.5	26.9 ± 23.8	31.3 ± 27.5
Cognition	38.5 ± 16.6	36.3 ± 15.8	44.5 ± 18.3	50.9 ± 20.4*	43.1 ± 15.8	40.6 ± 17.9	45.4 ± 21.5	45.1 ± 18.2
Communication	41.2 ± 19.8	46.2 ± 19.1*	56.2 ± 19.6 **	63.4 ± 17.7**	49.4 ± 19.5	51.1 ± 23.7	59.9 ± 22.9**	66.4 ± 22.5**
Body discomfort	47.8 ± 21.8	37.7 ± 19.9**	43.8 ± 21.2	43.1 ± 22.4*	51.8 ± 21.0	41.9 ± 23.3**	45.1 ± 22.5*	37.8 ± 20.7**
Hoehn and Yahr staging (range 0–5)^c^, Schwab and England scoring (range 0–100)^b^, and Beck Depression Inventory (range 0–63)^c^								
Hoehn and Yahr (off medication)	3.1 ± 0.9	2.7 ± 0.9**	3.3 ± 1.0	3.3 ± 1.1	3.3 ± 0.8	2.8 ± 0.8**	3.4 ± 1.0	3.7 ± 1.0
Hoehn and Yahr (on medication)	2.3 ± 0.7	2.2 ± 0.7	2.9 ± 1.1**	3.1 ± 1.2**	2.4 ± 0.4	2.4 ± 0.6	3.1 ± 1.0**	3.3 ± 1.1**
Schwab and England scoring (off medication)	52.0 ± 21.4	72.9 ± 19.3**	53.1 ± 21.5	54.5 ± 23.9	52.9 ± 18.5	70.4 ± 18.6**	51.8 ± 23.9	50.7 ± 26.5
Schwab and England scoring (on medication)	84.1 ± 10.6	87.3 ± 11.7*	70.3 ± 21.3**	63.4 ± 22.8**	82.9 ± 12.1	86.4 ± 10.8*	64.5 ± 24.3**	66.1 ± 27.3**
Beck Depression Inventory	10.7 ± 8.6	9.7 ± 7.4	11.5 ± 10.5	7.5 ± 7.8	10.4 ± 6.6	11.2 ± 8.0	10.4 ± 8.8	8.9 ± 8.8
Levodopa equivalent PD medication								
Levodopa equivalent PD medication (mg)	1,330 ± 586	1,058 ± 574**	1,099 ± 660**	978 ± 529**	1,283 ± 517	829 ± 474**	853 ± 480**	778 ± 490**

* Statistically significant changes from baseline to each time point within each group (*p* < 0.05).

** Highly statistically significant changes from baseline to each time point within each group (*p* < 0.001).

a Not statistically significant between groups at each time point or for the overall linear trend across time.

b Higher score indicates better functioning.

c Higher score indicates worse functioning.

Both targets allowed significant medication reductions over time from baseline. GPi patients had a reduction in LEDD from 1,330 (±586) at baseline to 1,058 (±574, *p* < 0.001) at 2 years, 1,099 (±660, *p* < 0.001) at 7 years, and 978 (±529, *p* < 0.001) at 10 years; STN patients went from 1,283 (±517) at baseline to 829 (±474, *p* < 0.001) at 2 years, 853 (±480, *p* < 0.001) at 7 years, and 778 (±490, *p* < 0.001) at 10 years. There was no target difference over time for the linear trends using the mixed effect model (*p* = 0.70), while there were some marginally significant differences between the two target groups compared to baseline at 2 years (*p* = 0.04) and at 7 years (*p* = 0.05), with more reduction in LEDD in the STN group.

### Cognitive outcomes

A detailed analysis of the results of cognitive assessments is beyond the scope of this report; however, the results from the Mattis Dementia Rating Scale (DRS) clearly depict further progression of cognitive impairment in PD in the long-term follow-up after DBS, regardless of target. Twenty-seven of the 117 study participants still participating at 7 years had an MDRS score below 100 (out of a total possible score of 144) [16/68 in the GPi group (23%) and 11/49 in the STN group (22%)]. At 10-year follow-up, 30 of the remaining 77 study participants scored in that range [20/49 in the GPi group (40%) and 10/28 in the STN group (35%)], with no difference in rate of low scores by surgical target.

### DBS stimulation parameters

At 10 years, the average stimulation parameters were 3.0 (±1.0) V, 83.7 (±29.5) μs, 154.5 (±26.3) Hz for patients with STN targets and 3.9 (±1.2) V, 99.5 (±38.7) μs, 153.7 (±24.9) Hz with GPi; these were similar to stimulation settings seen at 2 years for both targets.

### Adverse events

For the period of this long-term follow-up study, we reported only non-MedDRA coded major medical events captured on case report forms, which included information on device-related events, emergency room visits, hospitalizations, or other serious medical events that the investigator considered to be important (Table 3) between year 2 and year 10 visits. As expected, nearly all patients (*N* = 153) in the study required implantable pulse generator replacements at some point due to the neurostimulator batteries reaching the end of life. There were 12 events of device malfunction and 10 DBS device-related infections. Hospitalizations were common during this period, with 29% of these visits being related to DBS. There were 414 visits from 114 patients to the emergency room (64 with GPi and 50 with STN), but only 17 visits were related to DBS. Fall injuries were also common, with 151 events reported from 74 patients (44 for GPi and 30 for STN), with 1 reported due to DBS.

**Table 3:** Adverse events.

Event type	GPi (*N* = 86)		STN (*N* = 70)		Total (*N* = 156)	
	Events	Unique patients	Events	unique patients	Events	Unique patients
DBS IPG explant (due to end of life)	179	84	138	69	317	153
DBS IPG replacement	177	84	140	69	317	153
DBS malfunction	8	8	4	4	12	12
DBS infection	6	5	4	4	10	9
DBS other issue	3	3	1	1	4	4
Hospitalization	198	60	114	57	244	117
Due to DBS	35	17	36	20	71	37
ER room visits	246	64	168	50	414	114
Due to DBS	10	9	7	6	17	15
Fall injury	98	44	53	30	151	74
Due to DBS	1	1	0	0	1	1
Other serious medical issue	105	45	66	29	171	74

## Discussion

This is the longest follow-up study to date of an initially randomized cohort describing DBS outcomes comparing the two targets. DBS therapy has a significant and stable effect on some aspects of motor function over 10 years, regardless of the target. Both STN and GPi targeted DBS provided significant improvement in tremor and rigidity subscores in the off-medication state, but motor assessments in the on-medication/on-stimulation condition continued to show a decline. This is consistent with previous open-label very long-term trials of both STN and GPi DBS (14–16, 18–21, 23, 24). Notably, DBS of both targets reduced motor fluctuations and dyskinesia out to 10 years. Over the very long term, both targets also allowed for a significant reduction in dopaminergic medication use, with no target differences, despite shorter-term studies often reporting a greater reduction possible with STN DBS. It is possible that with advancing disease, many patients may have decreased tolerance to dopaminergic therapy, limiting its use, but nevertheless, they have persistently improved motor function relative to baseline in the off-medication state and as measured by motor diaries.

Not all aspects of motor symptoms improve to the same extent at 7 and 10 years with DBS. Long-term studies of STN DBS have reported improvements in tremor and rigidity that persist longer than bradykinesia (22) and less benefit to axial PD symptoms, including speech, posture, gait, and postural instability (12, 14–20). Our study confirms these findings and also reports a similar decline with GPi DBS (23, 24, 46). Effective treatment of axial PD symptoms remains a critical unmet need.

Unfortunately, patients in our study (many of whom were relatively advanced in their disease at the time of enrollment) did not experience a halt in disease progression, nor did DBS therapy substantially influence many non-motor domains in the very long term. Patients continued to experience deterioration and greater disability. Again, we did not see differences between the two targets, as both cohorts were significantly worse on indicators of quality of life at 7 and 10 years. Detailed neuropsychological testing data were limited in the long term but based on the limited analysis of MDRS outcomes and surrogate markers of cognitive decline that were available (UPDRS I and items on the PDQ-39), we saw a similar decline in cognition across targets.

Adverse events for the two targets were also similar. Between years 2 and 10, there were only nine DBS infections reported across all subjects, and DBS device malfunctions were also generally rare.

This long-term study has several limitations. Despite patients being recruited from a randomized controlled trial initially, this study re-enrolled only a subset, and therefore, it no longer serves as a randomized, controlled study. However, the two cohorts did demonstrate similar baseline characteristics. We also had a high dropout rate, which is not unexpected for such a long trial and in a progressive disease, but still resulted in a relatively small sample size at 10 years. Furthermore, compared to the originally enrolled whole study population, the patients we enrolled and retained in the extension study may have experienced a relatively greater benefit from DBS and were therefore able to travel and participate in the study. This is supported by a greater reduction in this study at 2 years in the motor UPDRS III scores compared to the primary study outcomes (33). Nevertheless, we did not see major differences in long-term outcomes between the two targets.

In conclusion, this longest follow-up study describing DBS outcomes in Parkinson’s disease by comparing the STN and GPi targets from an initial randomized trial contributes to the understanding of the natural history of PD treated with DBS in the very long term. Our findings confirm that DBS therapy, regardless of target, has a significant positive effect on some motor functions (notably tremor, rigidity, dyskinesia, and motor fluctuations) and has less of an impact on others (bradykinesia, axial signs) over 10 years. The initial advantage of STN over GPi in medication reduction was not seen in the long term. These study findings can help inform patients living with DBS or considering DBS therapy about what they can expect in the later stages of the disease, up to 10 years. DBS therapy changes the PD phenotype in the long term, given its powerful effect on some motor symptoms (47). This is remarkable given that PD is a progressive neurodegenerative disease. Some have suggested that current DBS therapy may influence disease progression; however, this remains controversial and unproven so far (48). The field of DBS since this study was conducted has experienced many technological advancements, including the introduction of multiple independent current controls, directional electrodes, a greater stimulation parameter space, image-guided programming, and adaptive DBS, all of which may lead to reduced side effects and better long-term outcomes (49). However, the continued deterioration of some motor and many non-motor domains and related progressive disability highlights the great need for other novel PD interventions and therapeutics for these aspects of PD.

## Data Availability

Raw data supporting the conclusions of this article will be made available by the authors, without undue reservation pending approval of data use agreement.

## Group member of CSP 468F Study Group

Hines VA Cooperative Studies Program Coordinating Center: Tammy Barnett, MA; Mary Biondic, BA; Kimberly Carlson, MS; Barbara Chris; Erin Kathleen Dursa, PhD; Joyce Gilroy; Kwan Hur, PhD; Michael J. Klein, BA; Ping Luo, PhD; Jan Motyka, BS; Unnati Patel, MPH; Domenic Reda, PhD; Nadia Sakla, MD; Charles Spanbauer, MS; Kelly Tir, BA; Yongliang Wei, MS. Pharmacy Coordinating Center: Sheila J. Campbell, BSBA, MBA; Crystal Harris, PharmD; Amanda Snodgrass, PharmD; Julie Thornton, BS. National Institute of Neurological Disorders and Stroke: Claudia Moy, PhD. Department of Veterans Affairs Cooperative Studies Program Central Office, VA Office of Research and Development: Grant D. Huang, MPH, PhD; Timothy O’Leary, MD, PhD. Executive Committee: Kenneth A. Follett, MD, PhD; Crystal Harris, PharmD; Michael J. Klein, BA; Elaine Lanier-Bohan, RN, MS; Ping Luo, PhD; William J. Marks Jr., MD; Claudia Moy, PhD; Unnati Patel, MPH; Domenic Reda, PhD; Johannes C. Rothlind, PhD; Amanda Snodgrass, PharmD; Matthew Stern, MD; Frances M. Weaver, PhD. Data Safety Monitoring Board: Roy Bakay, MD (Chairman); Rick Chappell, PhD; Robert Hart, MD; George McCabe, PhD; Margaret Schenkman, PhD. Study Monitors: Carol Fye, RPh, MS, CCRP; Sharon Matzek, MPH, CCRA. Site Investigators: Matthew Brodsky, MD; Jeff Bronstein, MD, PhD; Kathryn Chung, MD; John Duda, MD; Emad Farag, MD; Nicholas B. Galifianakis, MD, MPH; Graham Glass, MD; Kathryn Holloway, MD; Stacy Horn, DO; Jyh-Gong Hou, MD; Eugene C. Lai, MD, PhD; Jill Ostrem, MD; Aliya I. Sarwar, MD; Meredith Spindler, MD; Indu Subramanian, MD; Jayne R. Wilkinson, MD. Site Coordinators: Farah Atassi, MD, MPH; Shannon Donovan, MPAS, PA-C; Linda Fincher, RN, BSN; Susan Heath, RN, MS; Miriam Hirsch, MS, RN; Virginia Janovsky, RN, MN, MS; Elaine Lanier, RN, MS; Mary Lloyd, RN; Susan O’Connor, RN; Suzanne Reichwein; Norma I. Renteria, LPN; Holley Stone, RN; Robin Taylor, MSN, FNP; Monica Volz, RN, MS; Heidi Watson, BSN. Psychologists: Randall Coeshott, PhD; Johannes C. Rothlind, PhD.
